# One size doesn’t fit all: cross-sectional associations between neighborhood walkability, crime and physical activity depends on age and sex of residents

**DOI:** 10.1186/s12889-016-3959-z

**Published:** 2017-01-19

**Authors:** Andrea S. Richardson, Wendy M. Troxel, Madhumita B. Ghosh-Dastidar, Robin Beckman, Gerald P. Hunter, Amy S. DeSantis, Natalie Colabianchi, Tamara Dubowitz

**Affiliations:** 1 RAND Corporation, Health Division, 4570 Fifth Avenue, Pittsburgh, PA 15213 USA; 2 RAND Corporation, Health Division, Santa Monica, CA 90407-2138 USA; 3 University of Michigan, School of Kinesiology, Ann Arbor, MI 48109-2013 USA

**Keywords:** Physical activity, Environment, Low-income populations

## Abstract

**Background:**

Low-income African American adults are disproportionately affected by obesity and are also least likely to engage in recommended levels of physical activity (Flegal et al. JAMA 303(3):235-41, 2010; Tucker et al. Am J Prev Med 40(4):454-61, 2011). Moderate-to-vigorous physical activity (MVPA) is an important factor for weight management and control, as well as for reducing disease risk (Andersen et al. Lancet 368(9532):299-304, 2006; Boreham and Riddoch J Sports Sci 19(12):915-29, 2001; Carson et al. PLoS One 8(8):e71417, 2013). While neighborhood greenspace and walkability have been associated with increased MVPA, evidence also suggests that living in areas with high rates of crime limits MVPA. Few studies have examined to what extent the confluence of neighborhood greenspace, walkability and crime might impact MVPA in low-income African American adults nor how associations may vary by age and sex.

**Methods:**

In 2013 we collected self-reported data on demographics, functional limitations, objective measures of MVPA (accelerometry), neighborhood greenspace (geographic information system), and walkability (street audit) in 791 predominantly African-American adults (mean age 56 years) living in two United States (U.S.) low-income neighborhoods. We also acquired data from the City of Pittsburgh on all crime events within both neighborhoods. Exposure: To examine cross-sectional associations of neighborhood-related variables (i.e., neighborhood greenspace, walkability and crime) with MVPA, we used zero-inflated negative binomial regression models. Additionally, we examined potential interactions by age (over 65 years) and sex on relationships between neighborhood variables and MVPA.

**Results:**

Overall, residents engaged in very little to no MVPA regardless of where they lived. However, for women, but not men, under the age of 65 years, living in more walkable neighborhoods was associated with more time engaged in MVPA in (β = 0.55, *p* = 0.007) as compared to their counterparts living in less walkable areas. Women and men age 65 years and over spent very little time participating in MVPA regardless of neighborhood walkability. Neither greenspace nor crime was associated with MVPA in age-sex subgroups.

**Conclusions:**

Neighborhood walkability may play a stronger role on MVPA than accessible greenspace or crime in low-income urban communities. Walkability may differentially impact residents depending on their age and sex, which suggests tailoring public health policy design and implementation according to neighborhood demographics to improve activity for all.

**Electronic supplementary material:**

The online version of this article (doi:10.1186/s12889-016-3959-z) contains supplementary material, which is available to authorized users.

## Background

Since the mid-1980’s, obesity has increased dramatically across developed countries [1] with socioeconomically disadvantaged African American populations disproportionately affected [2–4]. Physical activity is considered crucial for weight loss and maintenance, and it has many health benefits, including improved cardiometabolic disease risk profiles [5–7], glucose metabolism [5, 8], and better functional health in older adults [9]. Indeed, the Physical Activity Guidelines recommend that adults do at least 150 min a week of moderate-intensity, or 75 min per week of vigorous-intensity aerobic physical activity, or an equivalent combination of moderate- and vigorous-intensity aerobic activity in order to achieve substantial health benefits [10]. Yet most Americans fall far short of achieving the recommended levels of physical activity and the groups of people with the highest rates of obesity, low-income, racial/ethnic minority women, and the elderly, are also the groups least like to achieve recommended levels of activity [11, 12].

Increasingly, policy efforts have focused on modifying neighborhoods with the goal of promoting physical activity. On one hand, structural characteristics of neighborhoods, including greenspaces and aesthetically pleasing environments such as tree-lined streets and parks, and high rates of walkability (i.e., environments that promote walking through sidewalks, traffic calming measures, etc.) may encourage physical activity and reduce obesity [13–16]. On the other hand, people living in socioeconomically deprived areas with high crime rates may worry about safety and limit their activity outdoors in spite of potentially high walkability or even greenspace [17–19] Therefore, policy makers need to understand which strategies may improve MVPA for people living in socioeconomically disadvantaged neighborhood where safety concerns may hinder physical activity [20, 21].

Still, neighborhood environments are complex and the role of objectively measured crime, combined with neighborhood walkability (e.g., pedestrian safety and mixed land use), and the concurrent role of these factors in influencing physical activity behaviors are not well understood (see reviews [22, 23]), especially in low-income African American populations [24, 25]. The overarching assumption is that increasing opportunities for physical activity in a neighborhood, both in terms of the structural characteristics and safety of the environment, will motivate residents to take advantage of the new opportunities and thereby increase their activity. However, increasing evidence (albeit mixed) suggests that associations between crime and neighborhood greenspace and walkability on physical activity differ according to age and sex, especially at older ages [17, 26–29] when physical activity declines [11, 30, 31]. For instance, among Canadian adults (mean age 41 years), women were more likely than men to feel unsafe, limit their walking and perceive less neighborhood walkability [27]. However, this study was based on a Canadian sample of relatively high-income (~60% with income > $60,000) white people that limits generalizability. In the U.S., a 7-year study of a large cohort of young ethnically diverse US adults (ages 11 to 29 years) showed that associations between self-reported frequent bouts of MVPA with objectively measured landscape diversity and lower crime rates are consistent in males and females [19]. In an older, more socioeconomically disadvantaged sample of 901 adults (mean age 45 years) living in 55 low-income District of Columbia neighborhoods, that included objective measures of crime and walkability, women were more likely than men to report less walking because of fear, but gender differences in fear between men and women shrank as neighborhood violence increased [29]. In one of the few studies that examined predictors of accelerometer-derived MVPA in older adults across multiple countries, perceived safety from crime, and lack of barriers to walking were positively associated with MVPA but only in older adults (>55 years) [17]. In sum, much of the evidence is limited by self-reported activity that is vulnerable to bias [11] and a dearth of literature that examines objectively measured environmental attributes and relationships in older low-income African Americans who are at greatest risk of low rates of MVPA [11, 12] and inactivity related diseases, such as obesity [32–34].

This cross-sectional study capitalizes on a cohort of predominantly African American, low-income residents living in two urban Pittsburgh neighborhoods, the Hill District and Homewood. The Hill District is predominantly African American with 45% of African American residents earning an income below the federal poverty line (U.S. Census Bureau). Nearly 20% of the Hill’s residents in the civilian labor force are unemployed (with many others under-employed) (U.S. Census Bureau), and 35.8% report no access to a vehicle. The population Homewood population is 90–97% African American and is comparable sociodemographically to the Hill District [35]. We examined the association between objective measures of the built and social environment and individual-level physical activity measured with accelerometers. We tested associations between neighborhood greenspace, walkability (e.g., pedestrian safety and mixed land use), crime and MVPA. We further examined whether these relationships were modified by age and sex. Consistent with prior literature, we hypothesized that neighborhood crime would play a stronger role on MVPA for women than for men [17, 27, 36] and that neighborhood walkability and greenspace would impact MVPA more so in younger than in older adults [11, 37].

## Methods

### Study population and participants

The data for the current study come from the Pittsburgh Research on Neighborhoods, Exercise and Health (the PHRESH Plus study) designed to document and evaluate neighborhood developments in greenspace and housing on physical activity and active transport in two lower-income African American neighborhoods in Pittsburgh, Pennsylvania. The sample includes randomly selected households from two communities. The households were originally selected from a stratified random sample of 2900 addresses zoned as residential from a list of addresses obtained after merging Allegheny County Office of Property Investment data with the Pittsburgh Neighborhood and Community Information System. Of the 1514 resulting addresses, we were able to contact 1259 (83%) of them. We found 1190 of these homes eligible for participation, and 1015 (88%) households completed the survey with an incentive. The Hill District (intervention) was scheduled to undergo various neighborhood revitalization initiatives, including renovation of greenspace for recreational activities. While some developments were planned for Homewood (control), they were not planned at nearly the capacity. This study uses the PHRESH Plus baseline data, (collected Spring 2013) prior to renovations. Data collection included a unique set of rich neighborhood-level built and social characteristics, in addition to detailed individual-level data. All study protocols were approved by the institution’s Institutional Review Board.

### Outcome variable: objectively measured individual-level physical activity

Participants were given a tri-axial accelerometer (i.e., Actigraph GT3X+) and asked to wear the device on their non-dominant wrist for 7 consecutive (24 h) days. Data were sampled at 30 hz and stored in gravity (g) units (1 g = 9.81 m/s). Data were processed in R using the GGIR package 1.2-8 (http://cran.r-project.org). Using static periods in the data, calibration error was estimated and corrected if necessary [38]. As has been done in other studies [39], nonwear time was identified when the standard deviation (SD) was less than 13 mg for 2 of the 3 axes or if the value range of each accelerometer axis was less than 150 mg, calculated for moving windows of 60 min with 15 min increments [40]. Acceleration was quantified using the vector magnitude from the three axes minus the value of gravity (g) (i.e., (x^2^ + y^2^ + z^2^)^1/2^ – 1) referred to as ENMO (Euclidean norm minus one). Negative numbers were rounded to zero. Minutes of moderate to vigorous activity were defined as a bout of at least 10 min of activity above the 100 mg threshold [41], where at least 80% of the bout was above the threshold of 100 mg. The average daily minutes of moderate to vigorous activity was calculated for those with valid wear time, which was defined as participants with at least 10 h of wear on at least 4 days.

### Exposure variables: neighborhood-level crime

Using incident-level crime data provided by the City of Pittsburgh police department, we calculated street network distances from each household to each crime location using ArcGIS 10.2. We were able to geocode 95% of the incidents using the address information from the raw data. We theorized that the crimes occurring in the preceding year would influence people’s perceptions of safety. Thus, for each household, we summed the number of crimes in calendar year 2012 that occurred within a 1 km network distance of the household address, to arrive at the household buffer for crime in 2012. We summed three types of crimes (property, violent, and sexual assault) that could impact participant’s decisions to be moderately-to-vigorously physically active in their neighborhood. Specifically, violent crimes were defined as incidents categorized as aggravated assault, homicide, and simple assault. Property crimes included incidents categorized as arson, burglary, motor vehicle theft, robbery, stolen property, theft, and vandalism. We used counts of crimes because in small-area studies, population-based rates can inflate the apparent threat from crime of an area [42].

### Neighborhood-level percent greenspace

While distance to urban greenspace is a common measure of access [15, 43, 44] we opted for a measure that would reflect the size of the land used for greenspace within a walkable area of the participant’s residence. [45–47] Specifically, we calculated greenspace area from the area surrounding each participant’s household within 1 km network buffer that was defined using the City of Pittsburgh’s GIS shapefile of Parks. The file was downloaded from http://pittsburghpa.gov/dcp/gis/gis-data-new, and the Parks data were last modified in October 2012. We divided the area of greenspace by the area within the buffer to create the percent greenspace.

### Neighborhood-level walkability

We derived the walkability index [48] from neighborhood audit data conducted on randomly selected street segments (both sides of a street between two cross streets) in both neighborhoods. Four trained data collectors walked the length of each segment between November and August 2013 to complete the audit, adapted from the Bridging the Gap Street Segment Tool [49, 50]. A member of our team was also a community member and neighborhood expert. She oversaw data collection, reviewed 10% of the sample to identify any and resolve any inconsistencies. We audited a 25% sample of street segments within a quarter mile of the participant’s residence [51]. The walkability index was designed based on the social-ecological model and evidence that sidewalks and other street characteristics were associated with physical activity/walking [52–54]. Specifically, the walkability index was composed of the following items: traffic signs at the intersection (4 items) pedestrian crossings (2 items), sidewalks (10 items), lighting (2 items), transit (2 items), and mixed use (2 items). For each street segment we summed the items and used the average across the street segments (Cronbach’s Alpha = 0.77) [49] and the scale ranges from 0 to 22, with higher scores indicating greater walkability.

### Individual-level covariates

Between May and October 2013 participants completed interviewer-administered questionnaires (Additional file 1) with questions about age (date of birth), gender, educational attainment (categorized into some college/bachelors degree or more versus less than college), car ownership or access to a car, and marital/cohabitation status (married or living with a partner versus living alone). We also included annual household income per $1000 and we imputed missing values with multiple imputation by chained equations. We did not include race/ethnicity as a control variable because the majority of the sample (92%) self-identified as Black or African American. To capture participant characteristics that could limit MVPA we used their responses to the question “Does your health limit you when walking one block?” which we dichotomized to “a little or a lot” versus “not at all” and their identification of being either a car owner or having access to a car (yes/no).

### Analytic sample

We excluded participants if they were missing accelerometry data (*n* = 165), had less than 4 valid (≥10 h) accelerometer wear days (*n* = 30), were missing neighborhood geographic information (*n* = 6), extreme statistical outlier (>3 SD from the mean) MVPA time (>330 min/day for a 76 year old woman, *n* = 1), missing percent greenspace (*n* = 4), missing walkability index (*n* = 54), missing marital status (*n* = 1). The final analytic sample included 791 adults who wore the accelerometer for an average of 6.9 ± 0.5 days. We calculated T-tests (continuous variables) and Chi-Square tests (categorical variables) to compare the covariates in the included versus excluded (*n* = 261) samples. The excluded were younger (mean age 52 years, *p* = 0.005).

## Statistical analyses

We performed descriptive analyses and multivariable models using Stata 13.0 (StataCorp, College Station, TX). We calculated means and standard deviations (continuous variables) and percentages (categorical variables) of individual-level – and neighborhood-level characteristics, stratified by sex and age (≥65 versus < 65 years).

### Main effect models

In a single model for the full sample and controlling for individual-level covariates (age, gender, educational attainment, marital/cohabitation status, annual household income per $1000, limited walking 1 block, and car ownership or access to a car), we simultaneously tested MVPA as a function of the neighborhood-level variables: 1) crime; 2) greenspace; and 3) walkability. We used a zero-inflated negative binomial (ZINB) model since 55% of the sample had no MVPA time per week. ZINB models simultaneously estimate the associations between the independent variables with MVPA and the odds of non-participation in MVPA. Vuong tests supported the need to use zero-inflated regression models versus standard negative binomial models [55].

### Interaction models

We dichotomized age by the senior citizen definition of 65 years and over versus less than 65 years. We conducted interaction analyses in four steps. First, using the same ZINB approach described above, we assessed 3 three-way interactions with age (≥65 versus < 65 years)-by-sex-by- each of the neighborhood-level variables: 1) crime; 2) greenspace; and 3) walkability. We controlled for the same covariates as in the main effect model. A significant three-way interaction test indicates that there exists at least one significant two-way interaction across levels of the third variable but it does not mean that both two-way interactions are significant. To facilitate interpretation of the significant three-way interactions, we conducted two separate Analysis of variance (ANOVA) models with two-way interactions (sex-by- neighborhood-level variable) stratified by age (≥65 versus < 65 years). We considered the three-way interaction statistically significant when the F-ratio was significant in one age group and not in the other; results were adjusted for multiple testing [56]. Third, for significant three-way interactions, we illustrated our findings by estimating and plotting the predicted number of MVPA minutes per day for the particular neighborhood variable by sex and stratified by age (≥65 versus < 65 years). Fourth, we used the post-estimation command ‘margins’ in Stata 13.0 (StataCorp, College Station, TX) to estimate the effect of the neighborhood variable (with a three-way interaction) on MVPA by age and sex.

## Results

As shown in Table 1, the PHRESH Plus cohort includes an older (mean age 56 years), low-income population, burdened with low mobility, who are largely living alone and who engage in very low levels of MVPA. About half of the 791 participants were women under 65 years of age (mean age 46 years), a quarter were women 65 and over (mean age 75 years), the next largest group, which constituted 16%, were men under age 65 years (mean age 51 years), and the smallest group (7%) were men over the age of 65 years (mean age 74 years). Within a 1 km buffer of our cohort, there were an average of 328 crimes. Both the Homewood and Hill District neighborhoods had little green-space (mean 3.4%) and low walkability as indicated by the consistently low walkability index of about 8 (on a scale ranging from 0 to 22, with higher scores indicating greater walkability).

**Table 1:** Individual- and neighborhood-level characteristics by age (<65 versus 65+ years) and sex, means (SD) presented for continuous variables and percentages presented for categorical variables

	Women <65 years	Women 65 years+	Men <65 years	Men 65 years+	Total
	*N* = 413	*N* = 197	*N* = 124	*N* = 57	*N* = 791
Individual-level characteristics					
Outcome					
Moderate-to-vigorous physical activity (minutes/day)^a^ - mean (SD)	5.0 (10.0)	1.2 (4.9)	14.8 (21.8)	3.0 (14.0)	5.9 (17.4)
Socioeconomics					
Age (years) - mean (SD)	46.6 (11.7)	74.6 (6.7)	50.4 (11.5)	73.5 (6.9)	56.1 (16.3)
Household annual income (per $1000) - mean (SD)	11.6 (13.4)	14.8 (11.5)	16.4 (14.2)	14.9 (12.3)	13.4 (13.1)
Education some college/bachelors versus less than college - %	46.9	37.6	51.2	31.6	44.1
Married or living with partner versus living alone - %	25.1	11.7	23.2	17.5	20.9
Mobility					
Any physical limitation walking 1 block - %	22.2	41.6	23.2	40.4	28.5
Car owner or access to a car - %	58.9	50.3	64.0	49.1	56.9
Neighborhood-level characteristics					
Crimes in 2012^b^- mean (SD)	317 (150)	344 (181)	338 (178)	328 (159)	328 (163)
Percent green space^c^ - mean (SD)	3.3 (2.4)	3.9 (4.2)	2.9 (1.8)	3.8 (4.1)	3.4 (3.0)
Walkability index^d^ - mean (SD)	8.0 (2.0)	8.3 (1.9)	7.7 (1.7)	8.2 (1.6)	8.0 (2.0)

^a^ Average time moderately-to-vigorously physically active based on 4–7 days of accelerometry data and a bout of at least 10 min of activity above the 100 mg threshold [41], where at least 80% of the bout was above the threshold of 100 mg

^b^ Counts of crimes in 2012 within 1 km network buffer of residence obtained from Pittsburgh Police Department

^c^ Percent green space defined by percent of area with green space within 1 km network buffer of residence

^d^ The walkability index [48] was composed of the following items: traffic signs at the intersection (4 points), pedestrian crossings (2 points), sidewalks (10 points), lighting (2 points), transit (2 points), and missed use (2 points). Items were summed for each street segment and the average of the summed items across the street segments constituted the walkability index. The index ranges from 0 to 22

### Main effect models

In our main effect model, neither neighborhood greenspace, walkability, nor incidents of crime were statistically significantly associated with MVPA in the full sample (Table 2).

**Table 2:** The main effect model of zero-inflated regression models^a^ of moderate-to-vigorous physical activity (minutes/day)^b^ as a function of percent green space, crime, and walkability index

	β (SE)	*P*
Neighborhood-level characteristics		
Crimes in 2012^c^	<0.01 (0.00)	0.53
Percent green space^d^	−1.97 (3.17)	0.54
Walkability index^e^	0.07 (0.04)	0.05
Individual-level covariates		
Age (years)	−0.10 (0.00)	0.05
**Male**	**0.89 (0.16)**	**0.00**
Household annual income (per $1000)	<0.01 (0.01)	0.61
Education some college/bachelors versus less than college	−0.31 (0.15)	0.05
Married or living with partner versus living alone	−0.15 (0.15)	0.32
**Any physical limitation walking 1 block**	**−0.62 (0.20)**	**<0.01**
Car owner or access to a car	−0.15 (0.16)	0.32

Bold indicates statistical significance at *p* < 0.05

^a^ Zero-inflated negative binomial regression with “any limit walking 1 block”, “age”, and “male” in the inflate statement. Estimates are not presented but all were statistically significant at *p* < 0.001

^b^ Average time moderately-to-vigorously physically active based on 4–7 days of accelerometry data and a bout of at least 10 min of activity above the 100 mg threshold [41], where at least 80% of the bout was above the threshold of 100 mg

^c^ Counts of crimes in 2012 within 1 km network buffer of residence obtained from Pittsburgh Police Department

^d^ Percent green space defined by percent of area with green space within 1 km network buffer of residence

^e^ The walkability index [48] was composed of the following items: traffic signs at the intersection (4 points), pedestrian crossings (2 points), sidewalks (10 points), lighting (2 points), transit (2 points), and missed use (2 points). Items were summed for each street segment and the average of the summed items across the street segments constituted the walkability index. The index ranges from 0 to 22

### Interaction models

Among the five interaction models of age (65 and over)-by-sex-by- each of the neighborhood variables, only 1 was statistically significant: age-by-sex-by-walkability. For parsimony, we only present estimates for the statistically significant interaction where neighborhood walkability was differentially associated with MVPA depending on age and sex (Table 3).

**Table 3:** Three-way interaction model^a^ of age, sex, and walkability index^b^ on moderate-to-vigorous physical activity (minutes/week)^c^

	β (SE)	*P*
Age (years)	−0.01 (0.01)	0.34
**Male**	**2.72 (0.75)**	**<0.01**
Age 65+ years	1.60 (1.32)	0.23
Male * age 65+ years	**−9.52 (2.30)**	**<0.01**
Male * walkability index	**−0.24 (0.09)**	**<0.01**
Age 65+ years * walkability index	−0.22 (0.16)	0.23
Male * age 65+ years * walkability index	**1.04 (0.28)**	**<0.01**

Bold indicates statistical significance at *p* < 0.05

^a^ Zero-inflated negative binomial regression with “any limit walking 1 block”, “age”, and “male” in the inflate statement. Estimates are not presented but all were statistically significant at *p* < 0.001. Controlling for neighborhood crime, household annual income (per $1000), any limit walking 1 block, education some college/bachelors versus less than college, married or living with partner versus living alone, and car owner or access to a car

^b^ The walkability index [48] was composed of the following items: traffic signs at the intersection (4 points), pedestrian crossings (2 points), sidewalks (10 points), lighting (2 points), transit (2 points), and missed use (2 points). Items were summed for each street segment and the average of the summed items across the street segments constituted the walkability index. The index ranges from 0 to 22

^c^ Average time moderately-to-vigorously physically active based on 4–7 days of accelerometry data and a bout of at least 10 min of activity above the 100 mg threshold [41], where at least 80% of the bout was above the threshold of 100 mg

**Fig. 1:**
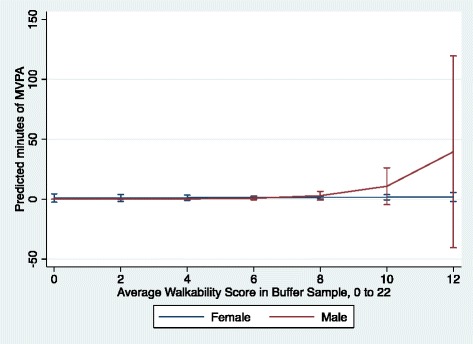
Model estimates of minutes of moderate-to-vigorous physical activity by walkability and sex for adults’ 65 + years, (*n* = 198 women and 57 men)

**Fig. 2:**
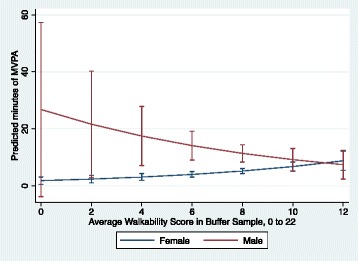
Model estimates of minutes of moderate-to-vigorous physical activity by walkability and sex for adults’ <65 years (*n* = 413 women and 124 men)

After correcting for multiple testing the critical *F*-value was 3.70, so a statistically significant F-ratio was > 3.70. Sex did not moderate the association between walkability and MVPA among those 65 and over (F-ratio = 3.64, df = 1) (Fig. 1). That is, women and men 65 years and over spent very little time participating in MVPA regardless of their neighborhood’s walkability. In contrast, among younger adults (<65 years), there was a significant sex by walkability interaction on MVPA (F-ratio = 5.91, df = 1) (Fig. 2). For women, but not men, under the age of 65 years, living in more walkable neighborhoods was associated with more time engaged in MVPA in (**β** = 0.55, *p* = 0.007) as compared to their counterparts living in less walkable areas (Table 4). For every one-unit increase in the walkability index, MVPA of women <65 years increased by 1.7 min per day (exponentiated value of 0.55) which translates to an increase from about 2 to 6 min as the walkability index increases from 0 to 12 (Fig. 2).

**Table 4:** Predicted slopes^a^ of walkability index^b^ on moderate-to-vigorous physical activity (minutes/week)^c^

	Walkability Index	
	Beta (SE)	*P*
**Women <65 years**	**0.69 (0.25)**	**<0.01**
Women 65 years+	−0.07 (0.35)	0.84
Men <65 years	−1.26 (0.91)	0.17
Men 65 years+	5.75 (6.83)	0.40

Bold indicates statistical significance at *p* < 0.05

^a^ Predictions based on zero-inflated negative binomial regression with “any limit walking 1 block”, “age”, and “male” in the inflate statement. Estimates are not presented but all were statistically significant at *p* < 0.001. Controlling for neighborhood greenspace, crime, household annual income (per $1000), any limit walking 1 block, education some college/bachelors versus less than college, married or living with partner versus living alone, and car owner or access to a car

^b^ The walkability index [48] was composed of the following items: traffic signs at the intersection (4 points), pedestrian crossings (2 points), sidewalks (10 points), lighting (2 points), transit (2 points), and missed use (2 points). Items were summed for each street segment and the average of the summed items across the street segments constituted the walkability index. The index ranges from 0 to 22

^c^ Average time moderately-to-vigorously physically active based on 4–7 days of accelerometry data and a bout of at least 10 min of activity above the 100 mg threshold [41], where at least 80% of the bout was above the threshold of 100 mg

## Discussion

This urban low-income and predominantly African American study population engaged in almost no MVPA. On average they engaged in about 6 min of MVPA per day or 42 min per week which is less than one third of the recommended levels of moderate intensity activity [10]. MVPA was even lower among women versus men and among those aged 65+ versus < 65 years. Lower rates of MVPA in older populations and women are consistent with findings from various countries where midlife and older adults accumulate 4–41 min of MVPA per day [30, 57–59]. Among a large sample of white and black adults (ages 49–99 years) living in the U.S. white and black women accumulated merely 3–4 min/day of MVPA [59]. One reason we observed such low MVPA rates in our population may be due to poor health that limits activity. On average, about 30% of our population reported that a physical limitation prevented them from walking one block. Even among the younger men who were about 46 years old, about 1 in 5 reported being physically limited. Interventions are critical to promote physical activity in underserved minority populations who have little time and resources, who are far less active than more advantaged populations [60–63], and who are burdened with more health limitations.

While policy efforts increasingly aim at community-level interventions to improve MVPA in disadvantaged neighborhoods, we found little evidence that the built and social environment impacted MVPA in this older adult, low income and predominantly African American cohort. Neither greenspace nor crime was associated with MVPA overall or in sex and age subgroups. The neighborhoods we studied had very little greenspace (approximately 3%), and that could have contributed to null findings. Compared to another study among New Zealand deprived neighborhoods that evaluated the proportion of usable greenspace in relation to obesity greenspace ranged only from 1 to 5% [64]. In addition to living near little greenspace, residents living in the Hill District and Homewood were also exposed to high crime rates. Based on Pittsburgh police data, the Hill District and Homewood typically have higher rates of violent crime, property crimes, and rape than other Pittsburgh neighborhoods [65]. The lack of associations we present here adds to the mixed literature. Despite researchers’ increased attention to the effects of neighborhood greenspace and crime on MVPA, findings were inconsistent and mixed across studies even when authors accounted for age and sex interactions [18, 28, 29, 66, 67]. However, ours was one of the few studies to examine objectively measured neighborhood walkability, greenspace, crime, and physical activity [68].

Consistent with our hypothesis, we observed a significant interaction between age, sex, and walkability, such that women aged <65 years living in walkable neighborhoods engaged in more MVPA than their older counterparts. While these results are encouraging, the effects appeared to be modest, such that the predicted amount of time spent in MVPA increased from 2 to 6 min/day in the most walkable surrounds among women < 65 years. The small effect may be due to poor walkability in these neighborhoods, with an average walkability index of 8 (max = 12) compared to the possible range of 0 to 22. However, our findings are comparable to the average walkability index of 6 that was reported in a national sample of public secondary school students and their communities [49]. The 4 min/day increase in MVPA (based on the difference in predicted 2 to 6 min of MVPA/day in Fig. 2) translates to a medium effect size of 0.3 (mean difference of 4 divided by standard deviation 12) [69] but increasing MVPA in sedentary adults is difficult. By comparison, an intensive 5-week intervention delivered face-to-face for 45 min for 18 inactive university employees randomly assigned to an intervention group to achieve 10,000 steps increased their daily MVPA from 20 to 35 min, an effect size of 1.1 [70]. While small changes in environment (often also more cost-effective relative to individual-level interventions) [71] may have a moderate effect on an individual they can have a significant population-level impact.

When examining neighborhood effects on health and health-related behaviors, important relationships could be missed when interactions are ignored and analyses pool across heterogeneous groups of people. For example, in a prior study, park proximity significantly interacted with retirement status such that non-retired participants who reported living near a park were more likely to participate in recreational walking, whereas no relationship was observed in retired participants [72].

We present data from a unique low-income and predominantly African American cohort living in underserved urban neighborhoods that includes a variety of detailed environmental data combined with individual-level characteristics and objectively measured MVPA. Accelerometry is superior to self-report where over-reporting can bias estimates [11]. Further, our study population are known to be at increased risk of residing in disadvantaged neighborhoods [73, 74], limited physical activity [11, 12] and suffering higher rates of inactivity-related cardiometabolic conditions [32–34]. To our knowledge, this is the first analysis linking objective measures of greenspace, walkability, and crime to accelerometry-derived activity in an older, disadvantaged, and predominantly African American population. Yet our study has some limitations. First, small sample sizes across age and sex subgroups may have limited our ability to detect associations between the neighborhood and MVPA, and with 3-way interactions by age and sex. Next, this study is cross-sectional and thus does not capture changes in the environment or MVPA. In addition, our 1 km network neighborhood buffer may not accurately reflect physical activity areas for different urban settings and sociodemographic subgroups. Indeed other walkability indices using geographic information systems data exist and are based on density, diversity, and design [75], such as the walkscore [76] that may capture a larger geographic area surrounding the participants’ homes. However, the quality of these measures depends on the accuracy of the data and they often include only limited snapshots in time that may not coincide with the study period. In contrast, a street audit is limited by a smaller sample than when using GIS but can collect more detailed information about street quality (e.g., lighting and sidewalk condition). We opted to collect street audit that coincided with the participants MVPA survey and MVPA data collection and provided a unique set of very detailed data that was collected through labor-intensive street audit. Further, participants excluded from this analysis were younger which may have biased our results. Residential location choice is complex and driven by more than physical activity preferences. Yet, individual physical activity may be tied to unobserved characteristics (e.g., health consciousness) that underlie an individual’s residential location. Thus residential selection could bias our results.

Despite these limitations, our study is an essential step in understanding how living in deprived neighborhoods with few greenspaces, low walkability and high crime rates may influence MVPA differentially across an older population of African American adults. Our findings are significant in light of the recent efforts to improve physical activity through policies targeting the built environment. Indeed, the campaign ‘Step It Up! The Surgeon General’s Call to Action to Promote Walking and Walkable Communities’ [77] recognizes that improving walkability through community design, transportation and land use is an important strategy to support American’s physical activity.

The need to improve low-resource neighborhoods is clear. In a large study including 7139 census tracts, comprising 9.5% of the 2010 US population, the availability of parks and recreational facilities was lower in predominantly minority census tracts relative to non-Hispanic white census tracts. [78] However, it may take more than small changes in the built environment to increase MVPA. The social component of choosing to use one’s neighborhood for MVPA needs to be considered. For example, social cohesion is a potent factor that influences people’s choices to use their neighborhood public space for physical activity [79]. Indeed, a community-level intervention aimed at improving the built environment in conjunction with a socially targeted program (e.g., family involvement) was more successful increasing physical activity in low-income neighborhoods than infrastructure interventions without a social component [80]. Greater understanding of how lifestyle factors and neighborhood greenspace, walkability and crime interact to influence MVPA is needed to inform effective policy.

## Conclusion

In sum, this study suggests that MVPA is strikingly low in middle-aged low-income African American adults and that neighborhood walkability may play a stronger role in MVPA than the proportion of accessible greenspace or crime in low-income urban communities. We found that walkability may differentially impact residents depending on their age and sex, which suggests a need for tailoring public health policy design and implementation to meet the diverse needs of residents based on neighborhood demographics.

## Additional file

Additional file 1: Questionnaire items. (DOCX 75 kb)

## Abbreviations

ANOVA: Analysis of variance
ENMO: Euclidean norm minus one
MVPA: Moderate-to-vigorous physical activity
PHRESH: Pittsburgh Research on Neighborhoods, Exercise and Health
U.S.: United States
ZINB: Zero-inflated negative binomial
