# Supplementary material for: One size doesn’t fit all: cross-sectional associations between neighborhood walkability, crime and physical activity depends on age and sex of residents
Source: BMC Public Health. 2017 Jan 19;17:97. doi: 10.1186/s12889-016-3959-z (PMC5248471; doi:10.1186/s12889-016-3959-z)
Supplement: Additional file 1: — Questionnaire items. (DOCX 75 kb) [file 12889_2016_3959_MOESM1_ESM.docx]

**[FILL IN FIRST ROW FOR RESPONDENT.]**

| **Age** | **Male/Female** |
| --- | --- |
|  |  |

What is your marital status?

**[CHOOSE ALL THAT APPLY]**

- Married (1)
- Living with a partner (2)
- Widowed (3)
- Divorced (4)
- Separated (5)
- Never married (6)
- DON’T KNOW (88)
- REFUSED (99)

What is the highest grade or year of school you completed?

- Never attended school or only attended kindergarten (1)
- Grades 1 - 8 (Elementary) (2)
- Grades 9 - 11 (some High School) (3)
- Grade 12 or GED (High School graduate) (4)
- 1 - 3 years of college (some college / technical school) (5)
- 4 years or more of college (college graduate) (6)
- Graduate School (7)
- DON’T KNOW (88)
- REFUSED (99)

What is your employment status?

- Employed full time (1) **→** **SKIP 8.3a and GO TO 8.4**
- Employed part time (2) **→** **SKIP 8.3a and GO TO 8.4**
- Not employed (3)
- DON’T KNOW (88) **→ SKIP 8.3a and GO TO 8.4**
- REFUSED (99) **→** **SKIP 8.3a and GO TO 8.4**

Do you own or lease a vehicle, such as a car, van, or truck?

- Yes (1) **→ SKIP 8.6 and GO TO 8.7**
- No (5)
- DON’T KNOW (88)
- REFUSED (99)

What was your total household income in 2012?

- Less than $5,000 (1)
- $5,000 - $9,999 (2)
- $10,000 - $14,999 (3)
- $15,000 - $19,999 (4)
- $20,000 - $29,999 (5)
- $30,000 - $39,999 (6)
- $40,000 - $49,999 (7)
- $50,000 - $59,999 (8)
- $60,000 - $74,999 (9)
- $75,000 - $100,000 (10)
- $100,000 or more (11)
- DON’T KNOW (88)
- REFUSED (99)

Does your health limit you a lot, a little, or not at all when ...

|  | **Limited a lot** | **Limited a little** | **Not limited** | **DON’T KNOW** | **REFUSED** |
| --- | --- | --- | --- | --- | --- |
| 1. Walking one block | (3) | (2) | (1) | (88) | (99) |
